# Dissolution of Lithium Contained in Lepidolite Using Ascorbic Acid: Kinetic and Modeling Analysis

**DOI:** 10.3390/ma17225447

**Published:** 2024-11-07

**Authors:** Sayra Ordoñez, Iván A. Reyes, Francisco Patiño, Hernán Islas, Martín Reyes, Miguel Pérez, Julio C. Juárez, Mizraim U. Flores

**Affiliations:** 1 Industrial Electromechanics Area, Technological University of Tulancingo, Tulancingo 43642, Mexico; sayraoh@hotmail.com; 2 Institute of Metallurgy, Autonomous University of San Luis Potosí, San Luis Potosí 78210, Mexico; alejandro.reyes@uaslp.mx; 3 CONAHCYT, National Council of Humanities, Sciences and Technologies, Mexico City 03940, Mexico; 4 Energy Engineering, Metropolitan Polytechnic University of Hidalgo, Tolcayuca 43860, Mexico; franciscopatinocardona@gmail.com (F.P.); islas_79hv@yahoo.com.mx (H.I.); 5 Academic Area of Earth Sciences and Materials, Autonomous University of Hidalgo State, Mineral de la Reforma 42183, Mexico; mreyes@uaeh.edu.mx (M.R.); miguel_perez5851@uaeh.edu.mx (M.P.); jcjuarez@uaeh.edu.mx (J.C.J.); 6 Higher School of Chemical Engineering and Extractive Industries, National Polytechnic Institute, Gustavo A. Madero, Mexico City 07738, Mexico

**Keywords:** lithium, lepidolite, characterization, decomposition, kinetics modeling

## Abstract

In this work, a kinetic study and modeling of the decomposition of a rock sample in an ascorbic acid medium with a high content of lepidolite phase were carried out, the results of which are of great importance due to the sample’s high lithium (Li) content. The rock sample was characterized by X-ray diffraction (XRD), inductively coupled plasma atomic emission spectroscopy (ICP-AES) and X-ray photoelectron spectroscopy (XPS), and the mineral species detected in the sample were lepidolite, at 65.3%, quartz, at 30.6%, and muscovite, at 4.1%, with a quantitative chemical analysis indicating the presence of elements such as Li, Si, K, Na, O, Al and, to a lesser extent, Fe and Ti; this highlights that the Li content present in the sample was 3.38%. Lithium was the element with which the chemical analysis of the kinetics was performed, resulting in decomposition curves comprising the induction period, progressive conversion and stabilization; this highlighted that the reaction progressed during the first two periods, obtaining a reaction order (*n*) of 0.4307 for the induction period and an activation energy (*Ea*) of 48.58 kJ mol^−1^, followed by a progressive conversion period with *n* = 0.309 and *Ea* = 25.161 kJ mol^−1^. This suggested a mixed control regime present in the lower temperature ranges, with a transition from chemical control to transport control present at high temperatures, with the study of the nature of the reaction and the concentration effect showing that chemical control predominates. The kinetic parameters and kinetic expressions for both periods were obtained, with the modeling showing that the calculated and experimental data do not present a major discrepancy.

## 1. Introduction

The growing demand for renewable energy and the global shift towards a low-carbon future has intensified the demand for critical energy elements such as lithium (Li) [1]. This element has become essential for the transition from fossil-fuel-dependent industries to cleaner [2] and green energy sources. In the coming decades, its sustainable production must be guaranteed [3,4]. There are 80 million tons of Li resources worldwide [5], with lithium being the 33rd most abundant element in the earth’s crust and ranging in quantity from 20 to 70 ppm. The United States Geological Survey (USGS) in 2022 showed that Australia (61,000 t), Chile (39,000 t) and China (19,000 t) are the world’s leading suppliers of Li, while Chile (9.30 Mt), Australia (6.20 Mt) and Argentina (2.70 Mt) have the largest quantities of Li in their reserves [6]. However, the growing demand for lithium has increased its price, and it is now sold as organic compounds containing chloride and bromide, such as butyl lithium [7,8,9], technical- and battery-grade lithium carbonate (Li_2_CO_3_) and lithium hydroxide monohydrate (LiOH·H_2_O), reaching a battery grade with a purity greater than 99% [3,10,11,12]. For example, the annual price of Li_2_CO_3_ in 2010 was USD 5180/t; in 2022, this increased to USD 37,000/t, increasing by seven times in twelve years [9]. The production of metal/li-ion substances (LIB) such as graphite, Li and cobalt will have to increase by 450% by 2050 [13,14]. At the pace of current demand, Li’s land reserve is expected to reach its limit in 2080 [9]. Most of the Li sources that would meet future demands are extracted from salt lakes, brines or seawater [15], which contain 66% of the world’s Li. Alternatively, reliable sources have emerged for the extraction of Li [16]. Because Li mines contain 34% of the world’s lithium [15,17], minerals such as ores and pegmatites [9], specifically spodumene and lepidolite [16,18], which are silicate minerals [19], are known as Li micas [20]; these contain Li_2_O contents of 8.03 and 7.7%, respectively [21]. In the case of an LiAl(SiO_3_)_2_ spur, calcination and acid roasting [22] is required to achieve a battery-grade purity greater than 99.5%, followed by the extraction of Li to produce Li_2_CO_3_ or LiOH·H_2_O [23,24,25,26]. Unlike the spur, the pretreatment of lepidolite K(Li_2_Al)(Si_4_O_10_)(F,OH)_2_ [27,28] is performed by coating with additives [29,30,31], obtaining leaching efficiencies of Li 97.7% and 95.9%, respectively. For this reason, an alternative hydrometallurgical route is sought for the extraction of Li [29,32], avoiding the use of inorganic acids (HCl, HNO_3_, HF or H_2_SO_4_) [33], their reaction with lepidolite and the generation of soluble Li salts, since the use of these acids generates a large amount of liquid pollutants and emits harmful gasses such as [34] Cl_2_, SO_3_ and NO_x_ that negatively affect the environment [35]. To reduce pollution during leaching, the use of soft organic acids such as citric, ascorbic, malic, oxalic, aspartic, succinic, etc., is proposed, adding H_2_O_2_ as a reducing agent; in addition, there are no studies on the decomposition kinetics of the organic acids of lepidolite. Therefore, this work focuses on developing synergistic research on lepidolite kinetics in an ascorbic acid medium, determining the nature of the reaction and evaluating the temperature, concentration and particle size effect when obtaining the activation energy and reaction order describing the process; this is followed by kinetic modeling, encouraging the contribution of research that provides an overview of new perspectives regarding the processing of alternative resources of Li [29].

## 2. Materials and Methods

To carry out the present work, a sample mineral with lepidolite as the majority phase was used; this sample was from the north of Mexico, Zacatecas state, the municipality of Fresnillo. The sample was pulverized with a porcelain mortar and subsequently sieved. Its mineralogical composition was determined by X-ray diffraction (XRD), (Bruker D8, Billerica, MA, USA, powder diffractometer, with Ni filtered radiation from a Cu anode; k_α1_ = 1.5406 Å; 40 kV and 35 mA; a recorded angular range of 2θ of 5–90°; step size = 0.01°; and step time = 3 s). The sample identification was performed using the software Diffract.Eva (Bruker) version 5.1 and using the ICDD-PDF-2 Release 2022 database. A quantitative chemical analysis was carried out using inductively coupled plasma atomic emission spectroscopy (ICP-AES, Perkin Elmer Optima 5300DV, Waltham, MA, USA), for which the mineral had to be dissolved with an acid solution of HCl, HF and H_2_O at a 1:1:1 ratio to determine the amounts of Li, Si, K and Al. Using atomic absorption spectroscopy (AAS, AAnalyst 200, Waltham, MA, USA, with Hollow Cathode Lamp), only the content of lithium was confirmed. For the analysis using X-ray photoelectron spectroscopy (XPS, Waltham, MA, USA), a solid sample was used and the quantities of each of the elements present in the mineral were determined. In addition, the conditions of use of the equipment were determined: the excitation source adopted an Al K–Alpha ray (hv = 1486.6 eV), the working voltage was 12.5 kV, the filament current was 16 mA and the signal was accumulated for 10 cycles. The analysis chamber vacuum degree was 8 × 10^−10^ Pa and the test passing energy was set to 100 eV).

For the kinetic decomposition study, a 500 mL glass reactor was placed on a magnetic stirring heating plate (500 rpm), and 0.5 g of lepidolite (mesh 400, 38 µm) was added to the reactor in a 0.057 M solution of ascorbic acid (C_6_H_8_O_6_). The temperature was varied (20, 30, 40, 50, 60, 70 °C) while the acid concentration (0.057 M) and particle size (38 µm) remained constant. To assess the impact of varying H_3_O^+^ concentrations (0.114, 0.085, 0.057, 0.028, 0.011, 0.003 M) on the system, the temperature (30 °C) and particle size (38 µm) were maintained constant.

To ascertain the impact of particle size, the concentration of H_3_O^+^ (0.057 M) and temperature were maintained at 30 °C, allowing for variation in particle size (53, 45, 38, 28, and 21 µm). During each experiment, 5 mL samples were taken and analyzed by ICP to determine the amount of lithium that had been leached. Two kinetic models were tested to determine which best fits the experimental data. The first was the product layer diffusion control mechanism (2/3 model), and the second was the surface reaction (1/3 model). The latter was determined to be the best fit, and thus was used for the entire kinetic study. To perform kinetic modeling, the values for the activation energy and reaction order for both the induction period and the progressive conversion period in the Arrhenius equation were replaced. The resulting data were graphed and calculated with the replacement.

## 3. Results and Discussion 

### 3.1. Mineral Characterization

**Figure 1:**
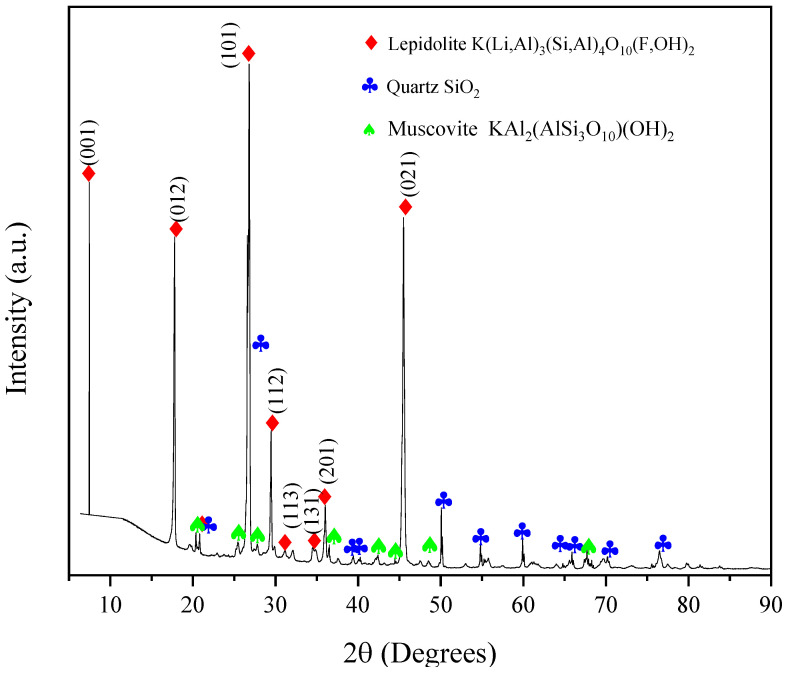
X-ray diffraction spectrum of the rock sample.

Figure 1 illustrates the X-ray diffraction spectrum of a rock sample composed of lepidolite (65.3%, 01-085-0398), quartz (30.6%, 00-046-1045) and muscovite (4.1%, 00-006-0263). The mineral phases that are consistently present with lepidolite are significant proportions of quartz and muscovite. However, in many instances, albite, hematite and thenardite may also be present, though in very low quantities. These last three minerals may be present in the rock, but they are undetectable by the spectrum.

Lepidolite is characterized by a tetrahedral–octahedral–tetrahedral (T-O-T) layer structure, whereby the tri-octahedral layer is situated between two tetrahedral leaves. The most prominent crystalline planes are the (001) at 7.54°, the (012) at 17.61°, the (101) at 26.80° and the (021) at 45.5°.

Once the mineral species present in the sample were identified, a quantitative chemical analysis was performed using XPS. This revealed the presence of Li, Si, K, Na, O and Al, as well as Fe and Ti, which may be present within the crystalline structure of lepidolite or muscovite. It should be noted that the high carbon content is due to sample preparation and adhesion with graphite tape.

Table 1 provides an overview of the elements present in the sample, the binding energy at which the signal begins, the binding energy at which the signal ends, the atomic percent present and the percentage by weight of each element present in the sample. The analysis indicates that the lithium content in the sample is 3.38%, which allows for the monitoring of chemical decomposition kinetics through the analysis of this element.

**Figure 2:**
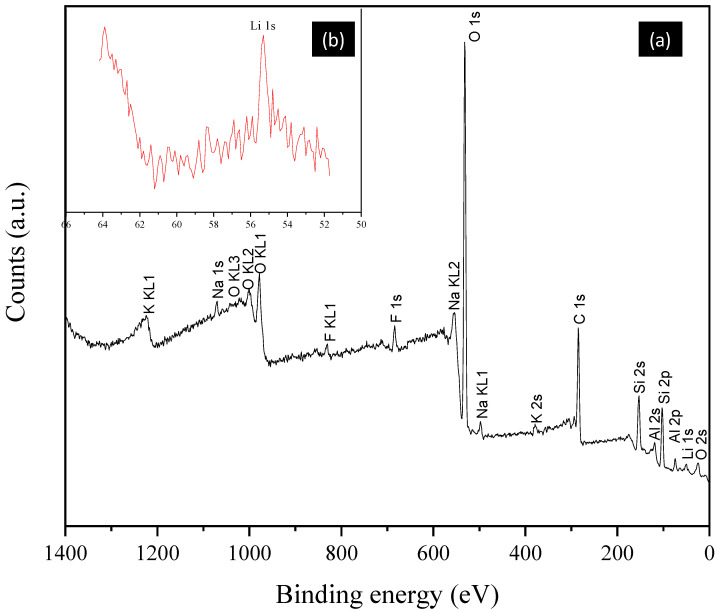
(**a**) XPS spectrum of the rock sample; (**b**) region of the spectrum where the lithium signal appears.

Figure 2a illustrates the spectrum of XPS. It shows that the Li-1s (Figure 2b) has a low binding energy (55.53 eV), and the peak intensity is relatively low due to the small amount present. The most abundant element is O-1s (531.97 eV), which is present in all oxides of the rock sample. It is worth noting that the sample was prepared in an environment contaminated by industrial waste. The spectrum displays two fluorine signals at 685 eV (F-1s) and 833 eV (F-Auger). This element is present in the chemical structure of lepidolite, as are Al-2s (71.41 eV) and K-2p (377.08 eV).

Table 2 presents the analytical results of the rock sample, which confirm the high content of Si, Al, Na and K. This can be attributed to the fact that the sample is mainly composed of silicates. The lepidolite formula includes potassium and aluminum, with sodium present due to impurities from other minerals, such as albite. Albite is commonly associated with this type of mineral, but its presence can be confused with quartz and lepidolite in X-ray diffraction.

### 3.2. Reaction Nature of Lepidolite in Ascorbic Acid

**Figure 3:**
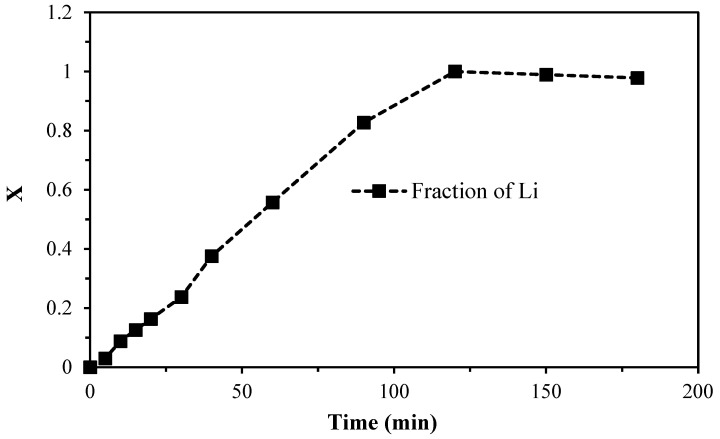
Reaction nature curve of lepidolite in ascorbic acid medium.

The primary objective of the reaction study is to ascertain the behavior of lepidolite in an ascorbic acid medium, with the data obtained from the mass fraction of lithium with respect to time, as illustrated in Table 3, serving as the basis for this investigation. Figure 3 illustrates the induction period, during which the reaction begins when the ascorbic acid solution comes into contact with the rock sample, resulting in the diffusion of hydronium ions (H_3_O^+^) from the solution while Li^+^ ions move from the particle’s exterior. This is followed by a period of progressive conversion, which lasts for approximately 15 min and continues for up to 90 min. Finally, the reaction reaches equilibrium, entering a stabilization phase. In addition to the lithium analysis, potassium and silicon were also analyzed. These elements did not react with ascorbic acid, as indicated by the ICP readings, which showed no significant changes in the solution.

During the progressive conversion period, the development of the reaction was evaluated. It was determined that the heterogeneous reaction model of spherical particles of constant size and an unreacted core is the most compatible. This model suggests that the reaction takes place first on the outer surface of the solid particle. Consequently, the solid reactant is converted continuously and progressively throughout the particle [36]. This model has two controlling stages: diffusion through the ash layer, which is called transport control, and chemical reaction control [37], as shown by Equations (1) and (2), respectively. In these equations, X is the mass fraction of decomposed lepidolite, *k_exp_* is the experimental rate constant and t is the time [37,38].

(1) $$1-3{\left(1-X\right)}^{2/3}+2\left(1-X\right)=k_{exp}t$$

(2) $$1-{\left(1-X\right)}^{1/3}=k_{exp}t$$

**Figure 4:**
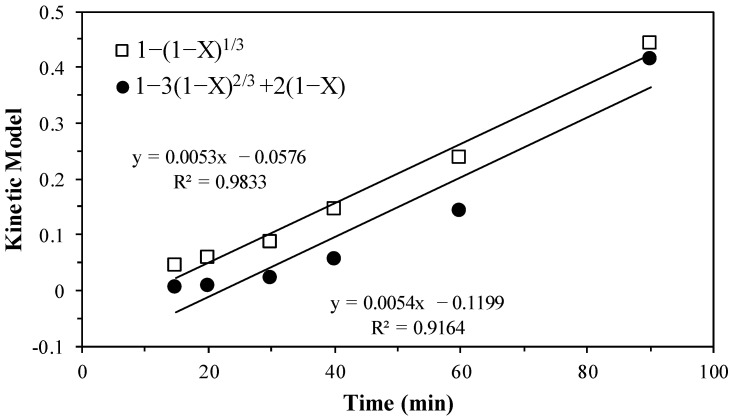
Evaluation of the progressive conversion period according to transport control and chemical reaction control.

Figure 4 illustrates the correlation between the kinetic model and time, demonstrating a notable 0.9833 correlation in the control by chemical reaction. This highlights that the reaction is promoted by the adsorption of ascorbic acid onto the interface of the ash layer. The chemical reaction occurs at the nucleus of the particle, resulting in the subsequent desorption of reaction products and the release of Li^+^ ions. It should be noted that the slow stage is the chemical reaction, which presents a greater resistance to the reaction rate. Therefore, Equation (3), which corresponds to the experimental rate constant, is used.

(3) $$k_{exp}=\frac{V_{m}k_{q}C_{A}^{n}}{r_{0}}$$

The molar volume of lepidolite (*V_m_*), with the formula K(Li,Al)_3_(Si,Al)_4_O_10_(F,OH)_2_, is 97.727 cm^3^·mol^−1^. Lepidolite is present in the sample with 65.3% of the material in question, as determined by the X-ray diffraction analysis. This figure is based on the quartz content, which is 30.6%, and the muscovite content, which is 4.1%. The molar volume was calculated using the molecular weight ratio (PM = 288.29455 g·mol^−1^) in regard to the density of the rock sample (ρ = 2.95 g·cm^−3^), where k_q_ represents the chemical rate constant, C_A_ denotes the concentration of the reactant [H_3_O^+^], n signifies the reaction order and r_0_ signifies the initial radius of the particle in micrometers (µm) [39]. 

### 3.3. Acid Decomposition Kinetics

The objective of conducting decomposition kinetics is to examine the factors influencing the reaction rate, including concentration, temperature, and particle size. As illustrated in Table 4, the concentration effect demonstrates a proportional increase in the rate *k_exp_*. Additionally, the induction time decreases with increasing ascorbic acid concentration, indicating that the reaction progresses more rapidly at higher concentrations. This results in a greater diffusion of hydronium ions [H_3_O^+^] with a minimum solution pH.

The rate of a chemical reaction is directly proportional to the concentration of the reactants. Therefore, to determine the rate of reaction, the sum of the exponent to which the concentrations of the reacting ascorbic acid molecules must be raised, which is known as the kinetic order of the reaction [40].

(4) $$logk_{exp}=log\frac{V_{m}k_{q}}{r_{0}}+nlogC_{A}$$

**Figure 5:**
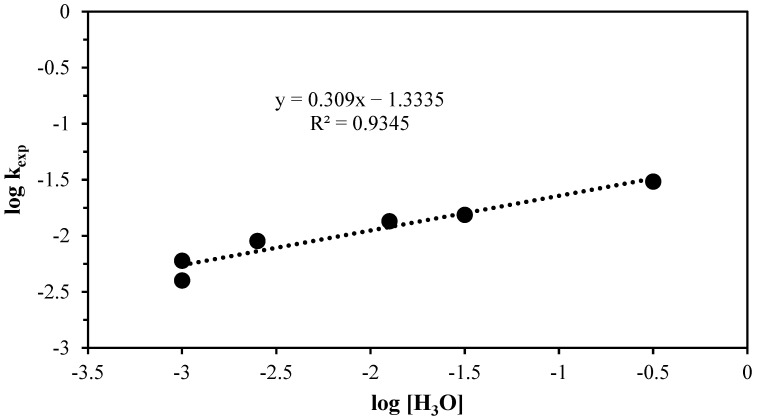
Graphical representation of ${log\;k}_{exp}$ vs. $log\left[H_{3}O^{+}\right]$ to obtain the reaction order in the progressive conversion period.

In light of the aforementioned context, it is possible to apply Equation (4) with logarithms from the experimental rate constant, given that the data for various values of ascorbic acid concentration are known. In Figure 5, the data referring to the progressive conversion period were evaluated, where the *log k_exp_* is dependent on the aforementioned variables, resulting in a linear relationship with a slope that represents the reaction order with respect to the ascorbic acid solution with varying concentrations. This yielded a value of 0.309. This result corroborates the study of the nature of the reaction, wherein the controlling stage is a chemical reaction that develops at a markedly slower pace compared to the phenomena associated with the transport of matter. There is no significant concentration gradient produced in the fluid film, indicating that the chemical rate constant is independent of the hydrodynamics of the process. This leads to a value of the reaction order that differs from the unit characteristics of the reactions in hydrometallurgy, which are typically associated with leaching using acids.

**Figure 6:**
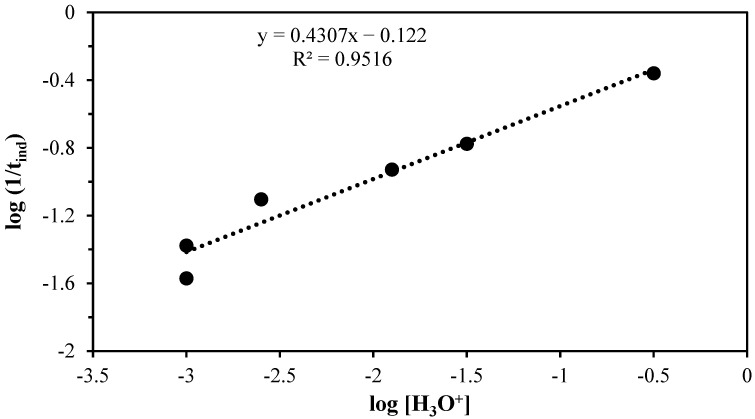
Graph of the induction period to obtain the reaction order.

Similarly, the reaction order for the induction period was obtained, as illustrated in Figure 6. This was determined through the graphical representation of *log* (1/*t_ind_*) vs. *log* [H_3_O^+^], which yielded *n* = 0.4. The data indicate a greater dependence on concentration during this period, which is part of the mechanism that controls the decomposition process. The formation of active points with a greater flow of hydronium ions [H_3_O^+^] gives rise to the development of the progressive conversion period.

While the reaction orders appear to be independent of hydrodynamics, they are in fact highly sensitive to temperature. This is demonstrated in Table 5, which shows that as the temperature of the ascorbic acid medium rises, the *k_exp_* increases, reducing the induction time and causing a greater flow of hydronium ions. [H_3_O^+^] implies the dissociation of the solution, which generates collisions that lead to a reaction. When molecules move faster, they collide with more force and more frequently, increasing the reaction rate. Consequently, the minimum activation energy (*E_a_*) is produced, which is required to start the chemical reaction. This can be deduced using Equation (5), which was formulated by Arrhenius [41,42].

(5) $$k_{q}=Ae^{{-E}_{a}/RT}$$

where $k_{q}$ is the chemical rate constant, A is the frequency factor, *E_a_* is the activation energy, *R* is the universal ideal gas constant (0.0083145 kJ mol^−1^ K^−1^) and T is the temperature in K [39,40].

(6) $$ln\frac{k_{exp}}{{\left[H_{3}O^{+}\right]}^{n}}=ln\frac{V_{m}A}{r_{0}}-\frac{E_{a}}{R}\left(\frac{1}{T}\right)$$

**Figure 7:**
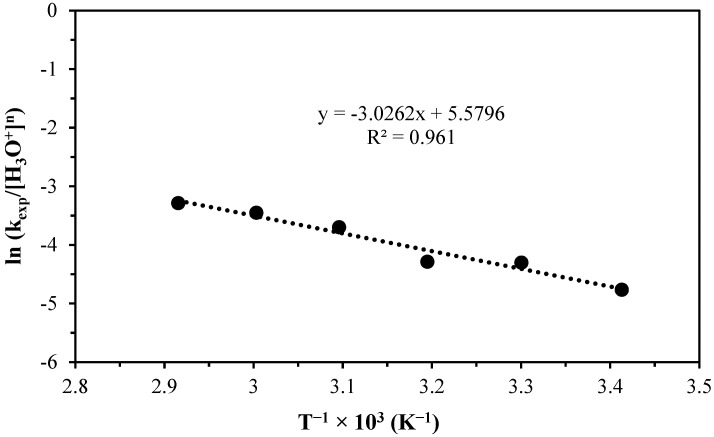
Graph of $lnk_{exp}/{\left[H_{3}O^{+}\right]}^{n}$ vs. $T^{-1}\times 10^{3}$ to obtain the activation energy in the progressive conversion period.

**Figure 8:**
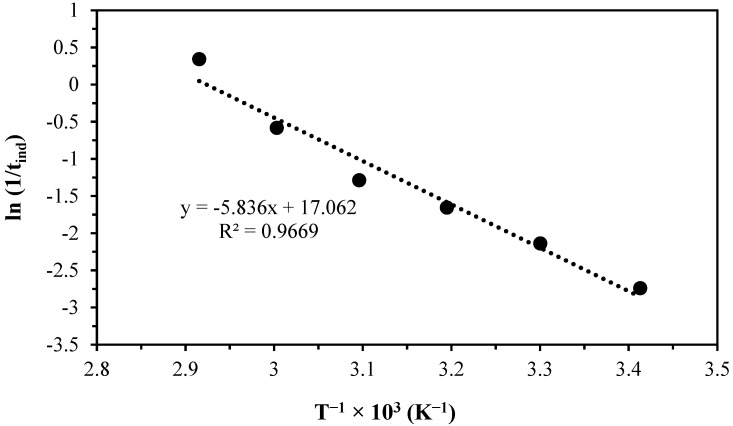
Graph of $ln\left(1/t_{ind}\right)$ vs. $T^{-1}\times 10^{3}$ to obtain the activation energy in the induction period.

By removing the pH variation with respect to temperature from Equation (4), due to the change in hydronium ion concentration [H_3_O^+^], we can apply the analogy *k_exp_/[H_3_ O^+^]^n^*. This results in Equation (6), which uses natural logarithms. The data for the conversion period are shown in Figure 7 as a function of the reciprocal of the temperature (T^−1^) × 10^3^), which is a straight line with a negative slope whose value is the ratio *E_a_/R* [37], allowed us to obtain an activation energy of 25.161 kJ mol^−1^ between 20 and 40 kJ mol^−1^. This indicates a mixed control regime, indicating that the transport of hydronium ions [H_3_O^+^] and the chemical reaction occur at comparable rates, with intermediate dependence on temperature and a moderate dependence on hydrodynamic variables. This shows that mixed control occurs at low temperature intervals with a transition of chemical control occurring at the interface of the unreacted core due to the transport control present at high temperatures. Figure 8 illustrates the relationship between *ln*(1/*t_ind_*) and *T*^−1^ × 10^3^. The straight line indicates a slope of 48.58 kJ mol^−1^, which suggests the presence of chemical control during the period of progressive conversion at low temperatures. Additionally, the frequency factor (*A*) was determined for both periods. This was calculated by extending and intercepting the straight line on the ordinate axis and raising it to the exponential, resulting in 2.57 × 10^7^ and 264.96 for the induction period and progressive conversion, respectively. This indicates that in the induction period, the frequency factor is higher due to the generation of more collisions, forming active points that exceed the origin of the progressive conversion period.

As shown in Table 6, a smaller particle diameter of lepidolite results in a higher reaction rate, leading to an increase in pH, *t_ind_* and the concentration of hydronium ions. This indicates that the expansion of the interface area corresponds to a proportional rise in the number of active points for the decomposition reaction [37].

**Figure 9:**
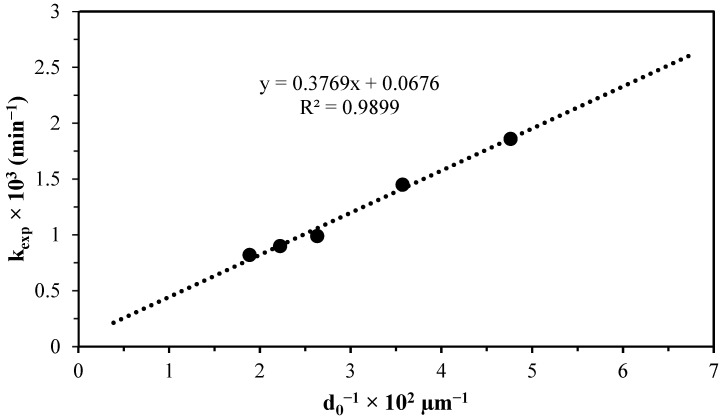
Graph of the progressive conversion period for the particle size effect on the decomposition of lepidolite in an ascorbic acid medium.

The particle diameter is evaluated as a function of the experimental constant for the progressive conversion period using Equation (3), as illustrated in Figure 9. The resulting straight line has a correlation coefficient of 0.9899, indicating a strong linear relationship between the experimental data points and the predicted values. The rate constant is inversely proportional to the particle diameter. Extending the straight line shows that it intersects the origin, indicating a characteristic chemical control regime for small particles where the product layer has small thicknesses [37]. This is in line with previous analyses.

**Figure 10:**
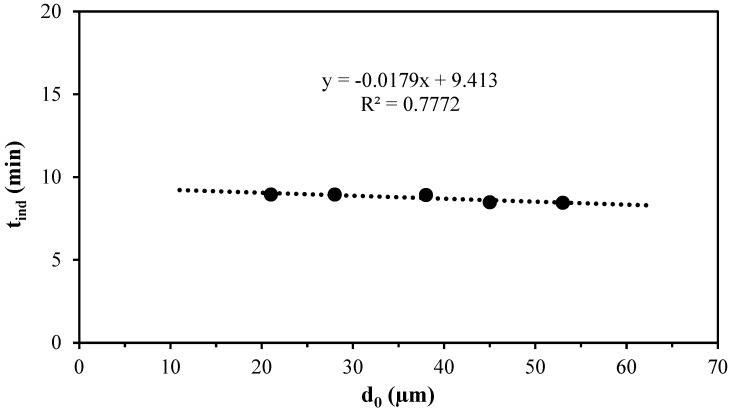
Graph of the induction period for the particle size effect on the decomposition of lepidolite in ascorbic acid medium.

Similarly, Figure 10 illustrates the correlation between the initial diameter of the lepidolite particle and the induction time. The graph displays a linear relationship, indicating that the particle size is not influenced by the induction time.

### 3.4. Kinetic Modeling

By studying the decomposition kinetics of lepidolite and considering the three effects analyzed, we derived Equation (7), which we call the general kinetic expression. This equation allows us to evaluate the data in Table 7. To derive it, we combined Equations (2), (3) and (5), which correspond to the controlling stage, experimental rate constant and Arrhenius, respectively.

(7) $$r_{Decomposition}=\left[1-{\left(1-X\right)}^{1/3}\right]=\frac{V_{m}}{r_{o}}Ae^{-E_{A}/RT}{\left[H_{3}O^{+}\right]}^{n}t$$

Equations (8) and (9) are kinetic expressions of the induction period and progressive conversion, respectively. They are derived from Equation (7) and involve the kinetic parameters that can be observed in the lower part of Table 7. These include the reaction order, the activation energy, and the frequency factor, which have been determined in the context of different effects.

(8) $$\frac{1}{t_{ind}}=\frac{1}{V_{m}r_{0}}{\left[H_{3}O^{+}\right]}^{0.4307}2.57\times 10^{7}e^{-48,580/RT}$$

(9) $${1-(1-X)}^{1/3}=264.96e^{-25,161/RT}{\left[H_{3}O^{+}\right]}^{0.309}t$$

**Figure 11:**
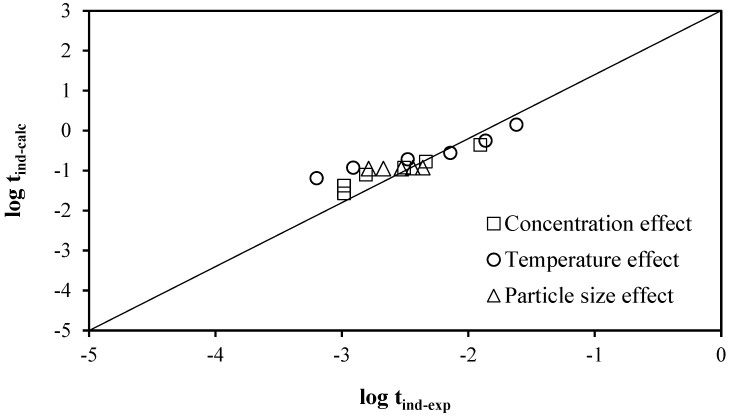
Plot of log_10_ of experimental induction period data vs. log_10_ of calculated induction period data for lepidolite in ascorbic acid medium.

**Figure 12:**
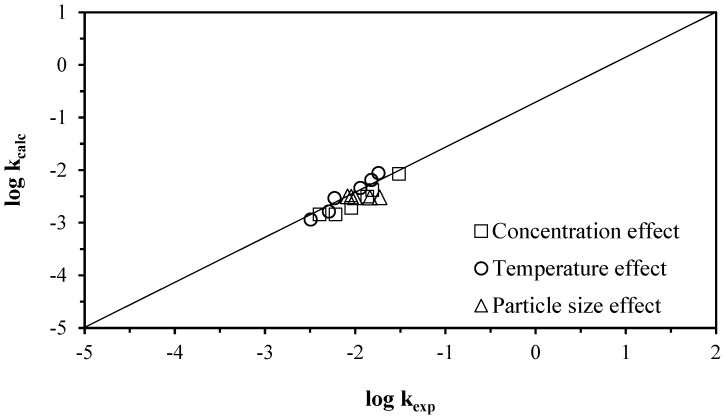
Graph of the log_10_ data for the experimental rate constant vs. the log_10_ constant calculated for the period of progressive conversion of the lepidolite in ascorbic acid medium.

By substituting the data in both equations, we can plot the graph of the experimental log_10_ induction time versus the calculated log_10_ induction time, as shown in Figure 11. Figure 12 illustrates the log_10_ of the experimental rate. The graph of the constant versus the log_10_ constant calculated for the progressive conversion period demonstrates that the data obtained from the kinetic expressions and the values obtained from the experimental process are similar. This indicates that the model accurately describes the decomposition of lepidolite.

**Figure 13:**
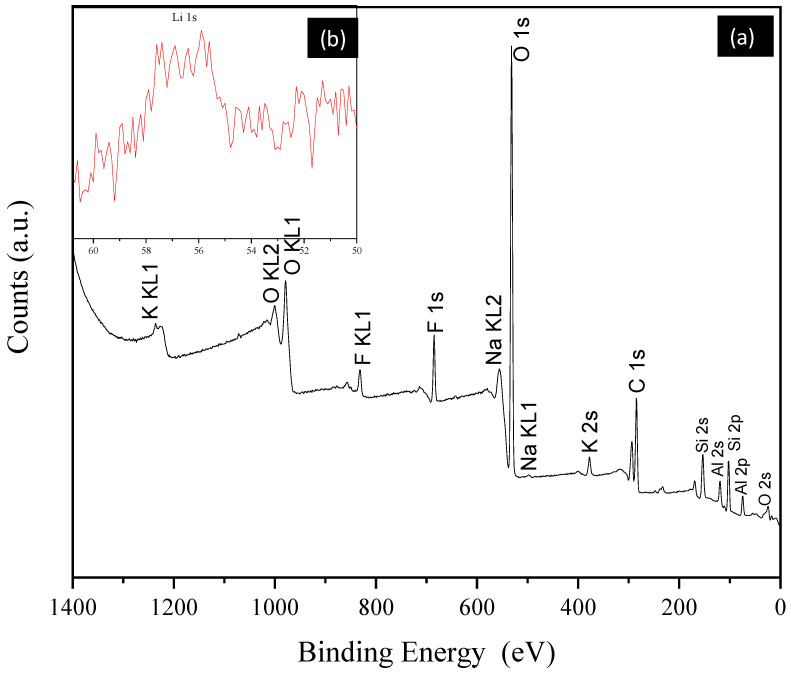
(**a**) XPS spectrum of the residues; (**b**) deconvolution of the region of the spectrum where the lithium signal occurs.

The dissolution residues were analyzed by XPS to determine which elements remain after this process. It was observed that the same elements continue to be present, but there is a decrease in the sodium and fluorine content. This can be observed in Figure 13a, which shows the results of the analysis of the dissolution residues, in comparison with Figure 2a, which shows the results of the analysis of the sample before the dissolution process. Figure 13b is the deconvolution of the area where lithium appears. It can be observed that the characteristic peak of this element no longer appears, and noise has entered this area. Therefore, it can be concluded that the dissolution of this element has been achieved.

### 3.5. Advantages of the Studied Method

The proposed method for dissolving lithium in a lepidolite mineral phase is environmentally friendly. The use of ascorbic acid is recommended, as it is considered non-polluting and has demonstrated a high dissolution efficiency. The activation energies for the induction period are 48.58 kJ mol^−1^ and for the progressive conversion period are 25.161 kJ mol^−1^, indicating a low dependence on the reaction medium. Previous work has used strong acids such as sulfuric acid, hydrochloric acid and hydrofluoric acid, among others. These acids are highly polluting and aggressive, which presents a public health risk. Using weak acids such as organic acids could be an ecological alternative for the treatment of minerals with high lithium contents. This work has demonstrated that ascorbic acid can be used in the processing of rocks with lepidolite mineral phases.

## 4. Conclusions

The rock sample was found to contain the lepidolite phase, with the formula of K(Li, Al)_3_(Si, Al)_4_O_10_(F,OH)_2_. This was determined through an X-ray diffraction study, which also revealed that the phase has a tetrahedral–octahedral–tetrahedral structure. The trioctahedral layer is located between two tetrahedral sheets. The rock sample contains 65.3 wt.% lepidolite, with a molar volume of 97.72 cm^3^ mol^−1^, along with other mineral phases, including quartz (30.6%) and muscovite (4.1%). The elements analyzed by ICP and XPS were identified as K, Na, O, Al, Fe and Ti, with silicon predominating. This is attributed to the minerals comprising silicates. Lepidolite contains 3.38% lithium, which diffuses into a solution of ascorbic acid. This reaction is controlled by chemical processes, with the lithium exiting the reaction slowly during the induction period, when active points are formed. The decomposition kinetics of lepidolite in an acid medium demonstrated an intermediate dependence on temperature and a moderate dependence on hydrodynamic variables. The kinetic parameters were determined to be *n* = 0.4307 with *E_a_* = 48.58 kJ mol^−1^ for the induction period and *n* = 0.309 with *E_a_* = 25.161 kJ mol^−1^ in the period of progressive conversion. During the conversion period, the process transitions from chemical control to transport control, with an activation energy ranging from 20 to 40 kJ mol^−1^. Kinetic modeling demonstrates that the proposed equations for both periods, with the corresponding kinetic parameters, align well with the experimental data within the chemical control model.

The use of ascorbic acid to dissolve lithium present in samples with lepidolite mineral phase represents an environmentally friendly alternative that can be employed at an industrial level. This acid is non-polluting, economical and demonstrates high dissolution efficiency. This lithium dissolution technique has the potential to be applied to mineral deposits that contain large amounts of lithium, given their efficiency in relatively short reaction times.

## Figures and Tables

**Table 1:** Elemental analysis by XPS of the lepidolite sample.

Element	Start BE ^1^ (eV)	Peak BE (eV)	End BE (eV)	Atomic %	PP At. %
Li 1s	60	55.53	51.73	3.38	3.22
Si 2p	108	102.76	98.44	17.12	19.33
C 1s	299.13	284.73	281	31.76	28.76
K 2s	391.10	377.08	359.52	0.95	1.86
Al 2p	75.3	71.41	68.24	0.05	0.15
O 1s	540	531.97	527.03	44.12	44.98
F 1s	691.82	685.71	673.21	1.23	1.30
Fe 2p	733.76	712.76	704.65	0.52	0.26
Na 1s	1077	1071.73	1067.48	0.87	0.14

^1^ Binding energy (BE).

**Table 2:** Chemical composition of rock sample.

Component	Li	Al	Si	Na	K	Fe	Mg
Weight (*w*/*w*)	3.76	8.82	39.64	5.31	4.94	0.12	1.02

**Table 3:** Time and mass fraction data from the study of the nature of the reaction of lepidolite in ascorbic acid.

Time (min)	Mass Fraction of Li
0	0
5	0.02967033
10	0.088111888
15	0.125974026
20	0.163036963
30	0.237262737
40	0.375624376
60	0.557442557
90	0.827172827
120	1
150	0.989010989
180	0.978021978

**Table 4:** Data on the concentration effect of lepidolite immersed in the ascorbic acid solution.

[C_6_H_8_O_6_] (mol L^−1^)	pH	[H_3_O^+^] (mol L^−1^)	*k_exp_* (min^−1^)	t_ind_ (min)
0.114	0.5	0.31623	0.0305	2.29
0.085	1.5	0.03162	0.0154	5.97
0.057	1.9	0.01259	0.0135	8.48
0.028	2.6	0.00251	0.009	12.7
0.011	4	0.00100	0.0060	23.82
0.003	6.1	0.00100	0.004	37.19

**Table 5:** $k_{exp}$ data for the temperature effect.

Temperature (°C/K)	pH	[H_3_O^+^] (mol L^−1^)	*k_exp_* (min^−1^)	t_ind_ (min)
20/293.15	2.84	0.00145	0.0032	15.48
30/303.15	2.83	0.00148	0.0051	8.48
40/313.15	2.45	0.00355	0.0059	5.23
50/323.15	2.25	0.00562	0.01139	3.62
60/333.15	2.15	0.00708	0.0151	1.79
70/343.15	2.1	0.00794	0.0181	0.71

**Table 6:** Data on the particle size effect of lepidolite in ascorbic acid medium.

Particle Size (µm)	Initial Ratio (µm)	pH	[H_3_O^+^] (mol L^−1^)	*k_exp_* (min^−1^)	t_ind_ (min)
53	26.5	1.89	0.01288	0.0082	8.45
45	22.5	1.91	0.01230	0.009	8.48
38	19	1.95	0.01122	0.0099	8.92
28	14	1.97	0.01071	0.0145	8.95
21	10.5	1.95	0.01122	0.0186	8.95

**Table 7:** Data corresponding to the study of lepidolite decomposition kinetics.

Effect	pH	[C_6_H_8_O_6_] (mol L^−1^)	[H_3_O] (mol L^−1^)		T K	d_0_ µm		T_ind_ (min)	*k_exp_* (min^−1^)
Concentration	0.5	0.114	0.31623		303.15	38		2.29	0.0305
	1.5	0.085	0.03162		303.15	38		5.97	0.0154
	1.9	0.057	0.01259		303.15	38		8.48	0.0135
	2.6	0.028	0.00251		303.15	38		12.7	0.009
	4	0.011	0.00100		303.15	38		23.82	0.0060
	6.1	0.003	0.00100		303.15	38		37.19	0.004
Temperature	2.84	0.057	0.00145		293.15	38		15.48	0.0032
	2.83	0.057	0.00148		303.15	38		8.48	0.0051
	2.45	0.057	0.00355		313.15	38		5.23	0.0059
	2.25	0.057	0.00562		323.15	38		3.62	0.01139
	2.15	0.057	0.00708		333.15	38		1.79	0.0151
	2.1	0.057	0.00794		343.15	38		0.71	0.0181
Particle size	1.89	0.057	0.01288		303.15	53		8.45	0.0082
	1.91	0.057	0.01230		303.15	45		8.48	0.009
	1.95	0.057	0.01122		303.15	38		8.92	0.0099
	1.97	0.057	0.01071		303.15	28		8.95	0.0145
	1.95	0.057	0.01122		303.15	21		8.95	0.0186
Induction period		n = 0.4307		E_a_ = 48,580 J mol^−1^			A = 2.57 × 10^7^		
Progressive conversion period		n = 0.309		E_a_ = 25,161 J mol^−1^			A = 264.96		

## Data Availability

The original contributions presented in the study are included in the article, further inquiries can be directed to the corresponding author.
